# Exploring the Relationships between Sex Hormones and Abdominal Muscle Area and Radiodensity in Postmenopausal Women: Insights from the Multi-Ethnic Study of Atherosclerosis

**DOI:** 10.1016/j.maturitas.2025.108197

**Published:** 2025-01-18

**Authors:** Amar Osmancevic, Matthew Allison, Iva Miljkovic, Chantal A. Vella, Pamela Ouyang, Penelope Trimpou, Bledar Daka

**Affiliations:** a General Practice/Family Medicine, School of Public Health and Community Medicine, Institute of Medicine, Sahlgrenska Academy, University of Gothenburg, Gothenburg, Sweden; b Division of Preventive Medicine, School of Medicine, UC San Diego, San Diego, CA, United States of America; c Department of Epidemiology, School of Public Health, University of Pittsburgh, Pittsburgh, PA, United States of America; d Department of Movement Sciences, University of Idaho, Moscow, ID, United States of America; e Institute for Clinical and Translational Research, Johns Hopkins University School of Medicine, Baltimore, MD, United States of America; f Department of Endocrinology, Institute of Medicine, Sahlgrenska Academy, University of Gothenburg and Sahlgrenska University Hospital, Gothenburg, Sweden

**Keywords:** Sex hormones, Sex hormone-binding globulin, Muscle composition, Post-menopausal women, Myosteatosis, Sarcopenia

## Abstract

The relationships between sex hormone levels and muscle composition in postmenopausal women remain underexplored. To address this gap, we conducted a cross-sectional observational study utilizing data from the Multi-Ethnic Study of Atherosclerosis. Our analysis included 682 postmenopausal women aged 45–84 years with complete data, with a mean age of 63.3 years. Using abdominal computed tomography, we assessed abdominal muscle area (cm^2^) and muscle radiodensity (Hounsfield units) in relation to serum levels of testosterone (total and free), estradiol, and sex hormone binding globulin (SHBG), measured in nmol/L.

Multivariable linear regression models, adjusting for potential confounders, were employed to investigate these associations. In our fully adjusted models, higher levels of estradiol and free testosterone were found to be positively associated with total area of abdominal muscle (β = 1.41, 95 % CI 0.4, 2.4, p = 0.007 and β = 18.5, 95 % CI 4.0, 33.1, p = 0.004, respectively), but not with muscle radiodensity (p > 0.05). Conversely, elevated levels of SHBG were associated with a smaller total of area abdominal muscle and radiodensity (β = −2.1, 95 % CI −3.2, −0.9, p = 0.001 and β = −0.32, 95 % CI −0.6, −0.0, p = 0.07, respectively).

Our study highlights significant associations between sex hormone levels and skeletal muscle area in post-menopausal women. Furthermore, the novel findings regarding SHBG and muscle composition suggest a potential previously unrecognized role of SHBG in the accumulation of skeletal muscle adipose tissue. However, further validation in other cohorts is necessary to elucidate the potential role of SHBG in body composition.

Clinical Trial: NCT00005487.

## 1. Introduction

Abdominal obesity is strongly associated with an elevated risk of cardiometabolic disorders and mortality [1]. Recent research has highlighted that both the quality and quantity of abdominal muscle plays an important role in cardiometabolic health [2]. The focus on abdominal muscle area in this study stems from its relevance to cardiometabolic health [3]. Studies using lumbar computed tomography (CT) scans have measured muscle quantity, defined as muscle area in cm^2^, and indirectly muscle quality, defined as muscle radiodensity in Hounsfield Units (HU). Lower muscle quantity (i.e. sarcopenia) and higher ectopic adipose accumulation in muscle (i.e. myosteatosis) have been associated with increased risk of all-cause mortality [2,4,5]. Conversely, higher levels of muscle radiodensity have been associated with increased physical strength/performance and cognitive function, further supporting the importance of maintaining muscle quality [6,7].

Women are at greater risk of cardiometabolic diseases after menopause, partly due to changes in body composition and in sex hormone levels [8,9]. Specifically, declining estrogen and changes in testosterone levels during the menopausal transition have been shown to affect muscle mass and strength, yet the exact mechanisms remain unclear [8,10]. While certain studies have reported positive impacts of estradiol on muscle strength, others found no significant associations [11]. Notably, a recent systematic review highlighted these knowledge gaps and emphasized the need for further research into the effects of endogenous estrogen on muscle composition in postmenopausal women.

Similarly, the relationship between endogenous testosterone levels and muscle mass in postmenopausal women is inconsistent. For example, five of six studies reported in a systematic review found no evidence for an association between total testosterone and muscle mass in women [12]. Interestingly, some studies investigating levels of free testosterone and muscle mass in postmenopausal women have reported significant positive relationships, while others have reported no significant relationships with muscle strength or performance [13,14]. Moreover, free testosterone has been shown to positively correlate with biopsy-derived type II (fast- twitch) muscle fibers after resistance training [15].

Finally, sex hormone binding globulin (SHBG), the main protein carrier of both testosterone and estradiol, may play an important role in muscle composition, but it remains underexplored [16]. Previously, we found a significant inverse association between SHBG and muscle radiodensity in men [17].

The aim of this study was to investigate the associations between fasting endogenous sex hormones, including SHBG, and abdominal muscle area and radiodensity in postmenopausal women.

## 2. Material and methods

### 2.1. Study design and study population

The current analysis is derived from data collected in the Multi-Ethnic Study of Atherosclerosis (MESA), a prospective cohort study that recruited 6814 men and women aged 45–84 years from six US communities (New York [NY], Baltimore [MD], Chicago [IL], Los Angeles [CA], Twin Cities [MI], and Winston-Salem [NC]) between 2000 and 2002. MESA participants were free of clinically diagnosed cardiovascular disease at baseline, and the study was designed to investigate the progression of subclinical cardiovascular disease over time. Six follow-up visits have been conducted since baseline.

For the current analysis, we used cross-sectional data from two MESA ancillary studies to investigate the associations between sex hormones and abdominal muscle area and radiodensity in postmenopausal women. More specifically, sex hormone levels were measured from baseline blood samples collected during the first MESA visit (2000–2002), while abdominal CT imaging data were collected on a random subset of 1970 participants (916 women) during the second or third MESA visits (2002–2005), with half of the participants having their scan during each visit. These assessments were not conducted concomitantly, with a time interval of 2–5 years between the hormone measurements and CT imaging. The initial focus of obtaining CT scans of the abdominal region was to quantify calcification in the abdominal aorta. Later, an ancillary study to utilize the same scans for measuring abdominal muscle composition was approved and funded. For this analysis, we included all women over 18 years of age with available data on sex hormone levels, menstrual status, and cardiometabolic disorders or lifestyle factors who underwent CT imaging. Women who were not menopausal or had missing information or samples were excluded from the study.

### 2.2. Study measures

Details regarding the methods used in the MESA cohort have been published [18]. In summary, centrally trained staff collected and processed venous blood, performed blood pressure measurements and conducted all interviews. Fasting blood samples underwent standard processing and were stored at −80 °C.

Information on lifestyle factors, medications and co-morbidities was collected through validated questionnaires/measurements [18]. Participants self-reported their race/ethnicity at baseline according to 2000 US Census criteria. Physical activity and sedentary behavior (measured in MET-hours/week) were assessed using a comprehensive, semi-quantitative questionnaire [19]. Current medication usage was determined through comprehensive interviews, during which participants were requested to bring their medication containers to the clinics. Hypertension was defined as a systolic blood pressure exceeding 140 mmHg and/or a diastolic pressure above 90 mmHg or the use a blood pressure lowering medication [20]. Diabetes mellitus was defined as self-reported diabetes, use of glucose lowering medications or American Diabetes Association fasting criteria (≥126 mg/dL) [21]. All participants with lipid-lowering medication or with lipid concentrations according to the National Cholesterol Education Panel Adult Treatment Panel III guidelines (defined as a total cholesterol/HDL-cholesterol ratio > 5.0), were defined as having dyslipidemia [22]. The measurement of high-sensitivity C-reactive protein (hsCRP), a marker of systemic inflammation, has previously been described [23]. Postmenopausal status was determined by self-report, with adjustments made based on age, hysterectomy, bilateral oophorectomy, and menopausal age [24].

### 2.3. Assessment of endogenous sex hormones

Participants adhered to a fasting period of 12 h and refrained from smoking and engaging in intense physical activity for 2 h prior to each examination. Blood samples were collected for fasting purposes between 0730 and 1030 h [25]. Subsequently, serum samples were obtained by centrifugation at either 2000 x*g* for 15 min or 3000 x g for 10 min and promptly stored at −70 °C. These samples were then dispatched to the University of Vermont for prolonged freezer storage, where they have remained untouched since the inception of the MESA study.

Serum hormone levels were determined from archived samples at the Sex Hormone Laboratory, located at the University of Massachusetts Medical Center in Worcester, Massachusetts. Total testosterone and dehydroepiandrosterone (DHEA) levels were determined using radioimmunoassay kits at baseline. Sex hormone binding globulin (SHBG) concentration was assessed through a Chemiluminescent enzyme immunometric assay (Immulite kits, Diagnostic Products Corporation, Los Angeles, CA). The overall coefficients of variation for total testosterone, DHEA and SHBG were 12.3 %, 11.2 % and 9.0 %, respectively. Estradiol was measured using an ultra-sensitive radioimmunoassay kit (Diagnostic System Laboratories, Webster, TX) with an overall coefficient of variation of 10.5 %. Free testosterone (nmol/L) was calculated according to the method of Södergård [26]. Quality control serum was obtained from a ~ 10 % blind pool. The quality control serum was sourced from a substantial reservoir, aliquoted into storage vials in a manner indistinguishable from MESA participants. The minimal detectable limit was 0.04 ng/mL (0.14 nmol/L) for testosterone, 2.5 pg/mL (9.2 pmol/L) for estradiol, and 0.02 nmol/L for SHBG. Twenty-eight women had values below the detection limit, resulting in missing data.

### 2.4. Assessment of abdominal muscle area and radiodensity

At two clinical sites (Northwestern University, University of California Los Angeles), an electron-beam CT scanner (Imatron C-150) was utilized, while remaining clinical sites (Columbia University, Wake Forest University, and University of Minnesota) employed multidetector CT scanners (Sensation 64 GE lightspeed, Siemens S4 Volume Zoom, and Siemens Sensation 16). A 35 cm field of view was employed for the analysis. Instances where cutoffs were observed necessitated the utilization of imputation techniques. These methods included duplicating values followed by repeat measure *t*-tests on a randomly selected subset, employing regression equations, and utilizing ruler lines to estimate the areas. Approximately half of the participants in this study underwent CT scans at visit two, and the remaining half at visit three.

Using a semi-automated approach, measurement of total muscle and adipose tissue were conducted using Medical Imaging Processing Analysis and Visualization (MIPAV) software version 4.1.2 (National Institutes of Health, Bethesda, Maryland). Abdominal tissue was categorized based on Hounsfield units (HU), where levels −190 to −30 HU were defined as adipose tissue, −30 to −0 HU defined as mixed connective tissue, and values between 0 and 100 HU defined as abdominal muscle tissue [27].

**Fig. 1.**
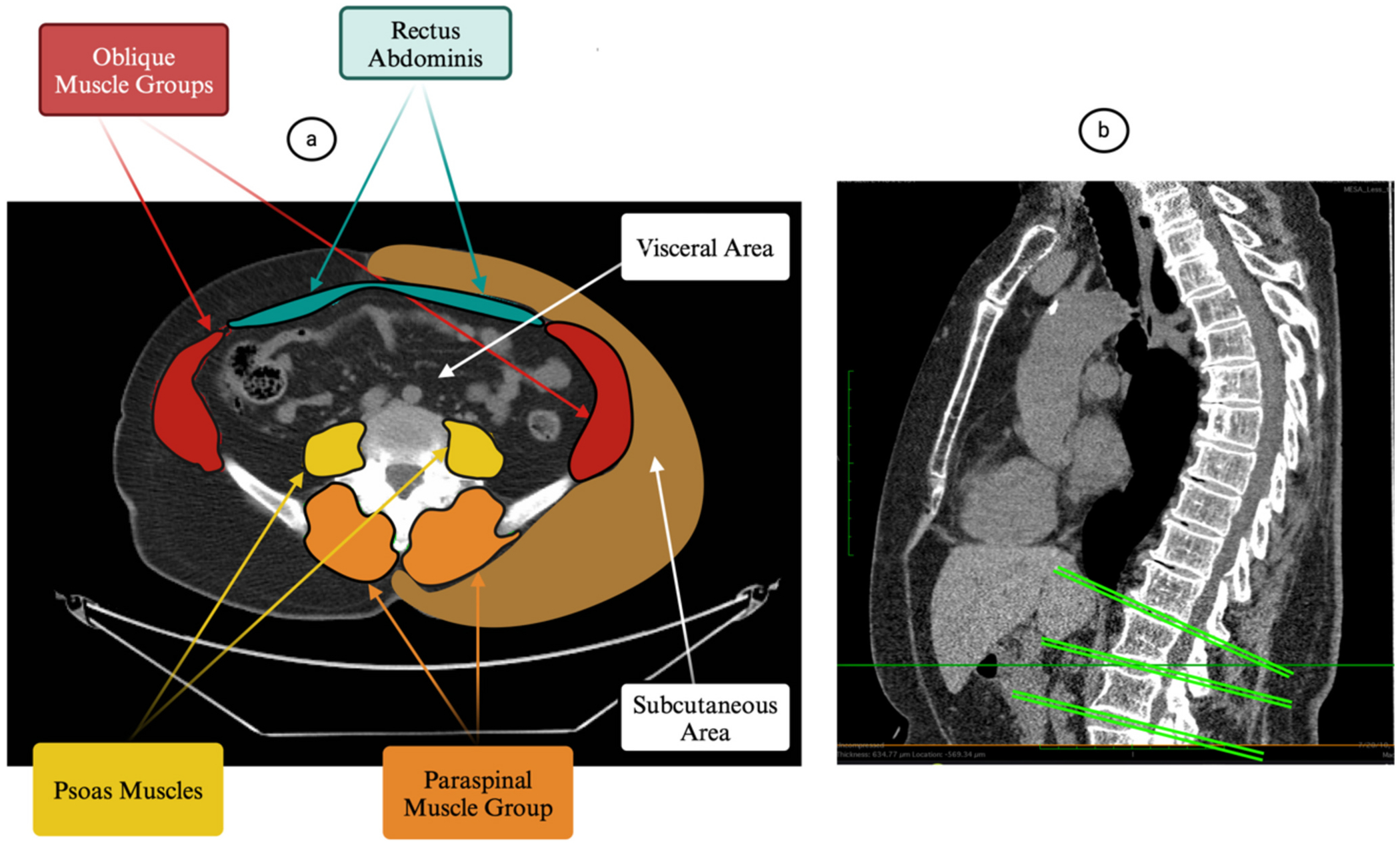
a) A cross-sectional image from the lumbar region displaying abdominal adipose and muscle tissue is presented. The categorization of abdominal tissue was based on Hounsfield units (HU), with values ranging from −190 to −30 HU designated as adipose tissue, −30 to 0 HU defined as mixed connective tissue, and 0 to 100 HU considered lean muscle. The areas of abdominal muscle and adipose tissue were computed by summing the pixel count, while muscle radiodensity was determined by the average HU value within the corresponding fascial plane of the muscle. Abdominal muscles were classified into stabilizing muscle groups (including the rectus abdominis, oblique muscle groups, and paraspinal muscles) and locomotor muscle groups (such as the psoas muscles). Subcutaneous adipose tissue was identified as adipose tissue located beneath the skin, while visceral adipose tissue referred to adipose tissue within the visceral cavity, excluding intermuscular adipose. b) A sagittal slice from the lumbar region is presented. Six transverse cross-section slices were analyzed: slice 0 corresponds to the L4/L5 vertebral junction, and slice 1 is immediately superior and adjacent to slice 0. Slice 2 is positioned at the L3/L4 junction with slice 3 superior and adjacent to slice 2. Slice 4 is located at the L2/L3 vertebral junction, and slice 5 is superior and adjacent to slice 4. CT scans were conducted with a collimation of 3 mm and a slice thickness of 6 mm.

Abdominal muscle area and adipose tissue area were calculated by summing the number of pixels within their average HU value, while abdominal muscle radiodensity was defined by average HU value within that muscle’s corresponding fascial plane. Four abdominal muscle groups – obliques, rectus, abdominis and paraspinalis (muscles of stabilization) and psoas (locomotor group) were assessed bilaterally (Fig. 1a). Total abdominal adipose tissue was determined as tissue within the HU range – 190 to −30 in the abdominal cavity [27]. CT scans were conducted with a collimation of 3 mm and a slice thickness of 6 mm. A total of six cross-sectional slices were captured at L2/L3, L3/L4 and L4/L5 intervertebral disc spaces (Fig. 1b) [5]. CT scans were configured with a collimation of 3 mm with a section thickness of 6 mm.

Research staff for assessing the CT scans were blinded to participants’ clinical information. The reliability of measurement, both inter- and intra-rate, for total abdominal area and all muscle groups was 0.99 and 0.93 to 0.98 respectively.

### 2.5. Statistical analysis

Continuous variables are shown as means and standard deviations (SD) while categorical variables are frequencies and percentages. Total, locomotor and stabilizing abdominal muscles presented normal distributions. Adjustments for body mass index (BMI, kg/m^2^) were applied for abdominal muscle areas, defining them as abdominal muscle indexes (total abdominal muscle area index (TAMAi) (TAMAi = TAMA/BMI), total stabilizing muscle area TSMA/BMI, and total locomotor muscle area index (TLMA/BMI)) [28].

Multivariable linear regression models were conducted to explore the relationships between sex hormone levels (total and free testosterone, estradiol, DHEA and SHBG) and abdominal muscle tissues. The outcome (measured as the standardized coefficient, β) was defined as the change in Hounsfield Units (HU) for muscle radiodensity and square centimeters (cm^2^) for abdominal muscle area for each 1-SD increment change in levels of testosterone (total and free), estradiol and SHBG. Model 1 adjusted for age, race/ethnicity, and level of education. Model 2 included variables from model 1 along with SHBG (no adjustment was made when investigating the associations of free testosterone and SHBG), and total abdominal adipose tissue. Model 3 incorporated variables from model 2 with additional adjustments for DHEA, CRP, physical activity, sedentary behavior, cigarette smoking, alcohol consumption, time from baseline to CT, years in menopause, hypertension, diabetes mellitus, dyslipidemia, exogenous estrogen use (excluding vaginal creams) and thyroid agents. The covariates in our models were assessed in baseline and selected based on their relevance to the relationship between sex hormones and muscle tissues. Age, race/ethnicity, and education level were adjusted for due to their influence on both hormone levels and health behaviors, which could affect muscle mass and body composition [29–31]. CRP was adjusted for because inflammation, as reflected by CRP, may affect muscle tissue and can interfere with hormone levels in postmenopausal women [32,33].

Our data fulfilled the criteria for linear regression analyses. We assessed multicollinearity in the linear regression analyses using the variance inflation factor (VIF), with no VIF exceeding 5 and the majority being around 1. No adjustment was made for estradiol for testosterone or vice versa due to their weak and statistically non-significant correlations (Pearson r = −0.027, p = 0.434; Spearman r = −0.005, p = 0.889), as this approach reduces the risk of over-adjustment. Models were also tested using BMI as a covariate. A two-tailed p-value <0.05 was deemed statistically significant. Analyses were conducted using IBM SPSS Statistics, version 29.

### 2.6. Ethical considerations

The MESA study protocol was approved by the Institutional Review Board at the Johns Hopkins University Hospital, University of California Los Angeles, University of Minnesota, Wake Forest University Hospital, Northwestern University Hospital, and Columbia University. All methods were performed in accordance with the relevant guidelines and regulations as set by the approving institutions in a standardized manner. Written informed consent was given by all participants [18].

## 3. Results

**Fig. 2.**
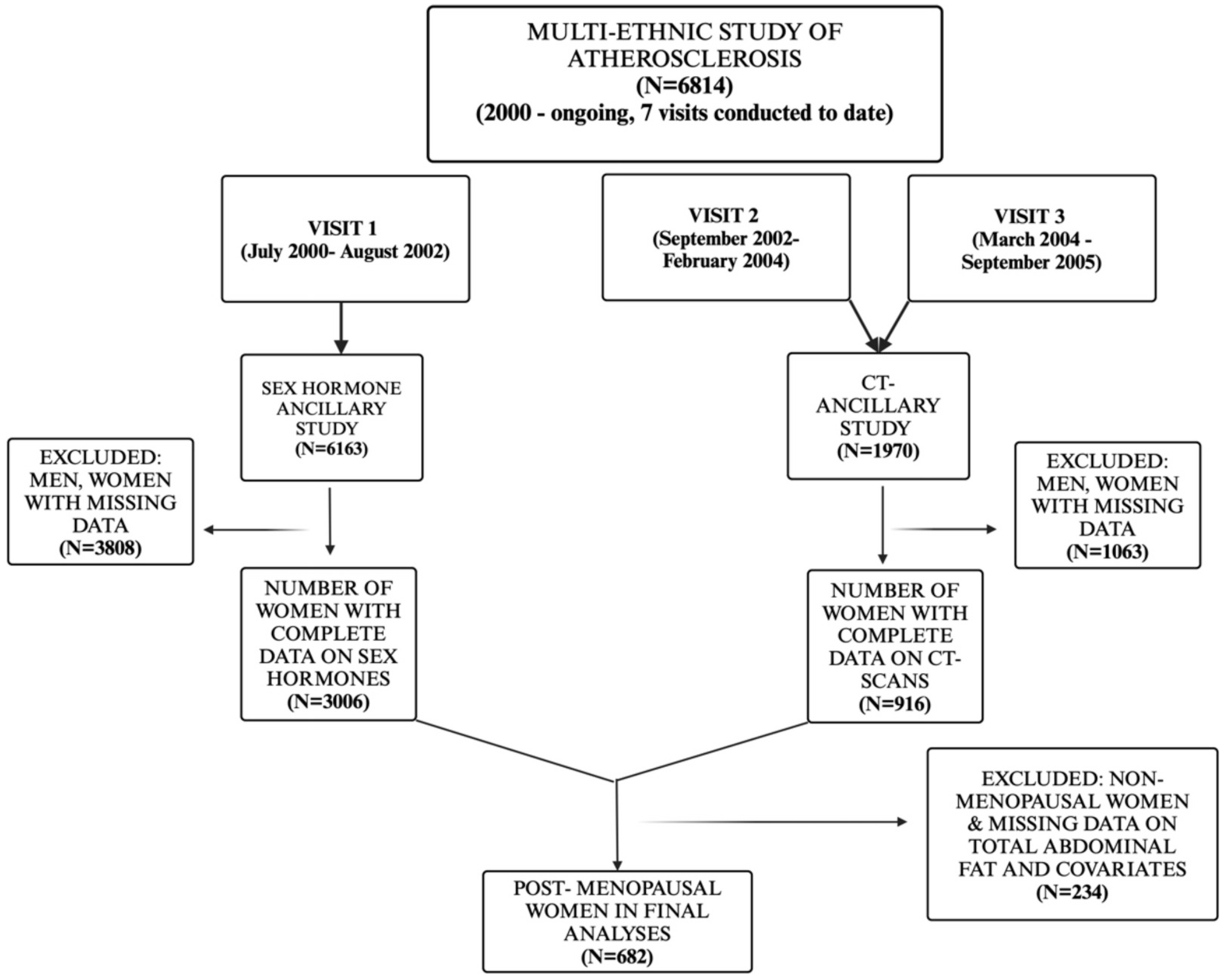
Flow-chart of the study population.

In this study, of the 6814 individuals in the MESA baseline examination, 3006 women with complete baseline data were identified. Among them, 916 women had complete data from the CT-ancillary study at visits 2 or 3. After further exclusions of non-menopausal women and those with missing data on total abdominal fat and covariates, the final sample consisted of 682 postmenopausal women (Fig. 2). There were no significant differences in baseline characteristics between the women included in the final analysis and those excluded, as well as compared to all women with data on sex hormones (data not shown). Table 1 displays the characteristics of the study population. The average age and BMI of the participants were 63.3 (± 10.0) years and 27.4 (±4.2) kg/m^2^, respectively. Most individuals were non-Hispanic White (39 %, n = 368), followed by Hispanic/Latino (24 %, n = 229), African American (23 %, n = 153) and Chinese American (19 %, n = 125). Additionally, 49 % of participants reported a diagnosis of hypertension (n = 368), 11 % reported current cigarette smoking (n = 115), 10 % had diabetes mellitus (n = 129), and 30 % had dyslipidemia (n = 213). Thirty three percent (n = 234) of individuals reported using exogenous estrogen. Sensitivity analyses revealed significant associations between estradiol levels and abdominal muscle area when individuals undergoing exogenous estrogen treatment were excluded (β = 4.1, 95 % CI: 1.1 to 7.0, p = 0.008). However, no significant association was found with abdominal muscle radiodensity (β = 0.60, 95 % CI: −0.3 to 1.5, p = 0.17).

### 3.1. Association between sex hormones and abdominal muscle area and indexes

In model 1, *estradiol* showed a non-significant association with *total* abdominal muscle area (β =0.73, 95 % CI -0.1, 1.5, p = 0.07), which was stronger and statistically significant with further adjustment (Model 2: β =1.75, 95 % CI 0.8, 2.7, p *<* 0.001; Model 3: β =1.41, 95 % CI 0.4, 2.4 p = 0.007) (Table 2) including total testosterone (β =1.40, 95 % CI 0.4, 2.4, p = 0.007). A significant association was also found between estradiol and stabilizing muscle area in final models (1.27, 95 % CI 0.4, 2.2, p = 0.005) but not for locomotor muscle area (p *>* 0.05). No significant associations were found for estradiol with total abdominal muscle area index (Table 3).

Higher levels of *total* testosterone were positively, but non-significantly, associated with higher total abdominal muscle area (Model 1; 5.99, −2.5, 14.5, p = 0.17 Model 2; 8.40, −3.9, 20.7, p = 0.18, Model 3; 11.26, −1.7, 24.2, p = 0.09). *Free* testosterone levels were significantly associated with higher abdominal muscle area in all models: Model 1: β =11.78, 95 % CI 1.6, 21.9, p = 0.02; Model 2: β =21.7, 95 % CI 8.7, 34.8, p = 0.001; and Model 3: β =18.5, 95 % CI 4.0, 33.1, p = 0.004 (Table 2). This association remained significant after additional adjustment for estradiol (β =19.5, 5.4, 33.5, p = 0.007). No significant associations were found for testosterone (*total and free*) or *estradiol* with total abdominal muscle area index (Table 3).

Interestingly, higher levels of *SHBG* were significantly associated with lower levels of *total* abdominal muscle area (Model 1; β = −1.46, 95 % CI -2.3, −0.6, <0.001, Model 2; β = −1.89, 95 % CI -2.8, −1.0, p < 0.001, Model 3; β = −2.10, 95 % CI -3.2, −0.9, p < 0.001) (Table 2), which remained significant after adjustment for both *total* testosterone and estradiol (−2.51, −3.7, −1.3, p < 0.001). A significant association was also found for *SHBG* with *total* abdominal muscle index in Model 1 (β =0.07, 95 % CI 0.0, 0.1, p < 0.001), which became non-significant with further adjustment (Model 2; β = −0.02, 95 % CI -0.1, 0.0, p = 0.19, Model 3; β = −0.03, 95 % CI -0.1, 0.0, p = 0.30) (Table 3). However, significant associations were observed when BMI was included as a covariate (data not shown). No significant associations were observed between DHEA and abdominal muscle composition (data not shown). Furthermore, the association between SHBG and Estradiol remained statistically significant when adjustment was made for BMI (−2.0, −3.5, −0.6, 0.006; 2.0, 0.6, 3.4, 0.005).

### 3.2. Associations between sex hormones and abdominal muscle radiodensity

*Estradiol* was non-significantly and inversely associated with *total* abdominal muscle radiodensity in model 1 (β = −0.1, 95 % CI -0.3, 0.1, p = 0.46). However, with further adjustment, the relationship between *estradiol* and *total abdominal* muscle radiodensity inverted but remained non-significant (Model 2; β =0.12, 95 % CI -0.1, 0.4, p = 0.36, Model 3; β =0.03, 95 % CI -0.3, 0.3, p = 0.98). This same pattern was found for both *total* and *free* testosterone and *total* abdominal muscle radiodensity (Table 4).

Intriguingly, the relationship between *SHBG* and *total* abdominal muscle radiodensity showed a positive, though non-significant, association in model 1 (β =0.14, 95 % CI -0.1, 0.4, p = 0.27), while further adjustment resulted in an inverse association in model 2 (β = −0.22, 95 % CI -0.5, −0.0, p = 0.09) and showed significant association in model 3 (β = −0.32, 95 % CI -0.6, −0.0, p = 0.04) (Table 4). This association remained similar, although non-significant, after adjustment for estradiol and *total* testosterone (β = −0.32, 95 % CI -0.6, 0.0, p = 0.06). Similar results were found for sex hormones, and SHBG with abdominal *stabilizing* and *locomotor* muscles (Supplementary tables). Significant negative correlations were presented between total abdominal adipose and total testosterone (r = −0.22, p < 0.001), and SHBG (−0.13, p < 0.001) and total abdominal muscle radiodensity (r = −0.36, p < 0.001). A statistically non-significant correlation with total abdominal muscle area (r = −0.19, p = 0.45). Moreover, a statistically significant and positive association was found between total abdominal adipose and estradiol (r = 0.07, p = 0.007). No significant differences were seen when adjustment was made for BMI except that the negative association between SHBG and abdominal muscle radiodensity became statistically non-significant (−0.23, −0.5, 0.07, 0.13).

## 4. Discussion

This study of a multi-ethnic cohort provides valuable insights into the significant associations between levels of sex hormones, SHBG, and abdominal muscle characteristics in postmenopausal women. Specifically, higher *estradiol* and *free* testosterone were significantly associated with higher total abdominal muscle area, while higher *sex hormone-binding globulin* was associated with lower levels of both total abdominal muscle area and radiodensity. These findings highlight the complexity of hormone interaction and their potential associations in muscle health among postmenopausal women.

Our study demonstrated significant associations between endogenous estradiol levels and abdominal muscle volume, particularly among women not receiving exogenous estrogen therapy. This would suggest that endogenous estradiol may play a protective role in preserving muscle mass in postmenopausal women. However, results in the literature have been inconsistent. While studies have shown that postmenopausal women generally showed reduced muscle mass compared to premenopausal women, the role of estrogen therapy in mitigating muscle mass loss was inconsistent [34]. In a recent review by Critchlow et al., studies reported inconsistent results, with some studies reporting beneficial role of estrogen and maintenance of muscle mass in post-menopausal women [16]. They proposed that the decline in estradiol levels might contribute to loss of muscle mass during menopause, but that its significance weakened after menopause. Interestingly, hormone replacement therapy with estradiol has been associated with reduced skeletal muscle breakdown in women within the first six years postmenopause, but this is not evident in late- menopausal menopause. Yet, in our study, the mean self-reported age of menopause was 47.8 years while mean age of the population was 63.3 years. This could suggest that maintaining normal estradiol levels over time may be important for muscle preservation. However, it is important to recognize that estrone (E1) becomes the predominant estrogen in postmenopausal women, which may help explain the inconsistent results observed between estradiol and muscle tissue decline in this population. Earlier studies have shown that estradiol affects muscle mass through different pathways. Animal studies have demonstrated that estrogen interacts with various signaling pathways, including those involved in apoptotic signaling, modifications of contractile proteins, and the maintenance of muscle satellite cells [35–37]. This crosstalk is crucial for understanding how estrogen influences muscle health and regeneration. Yet it has been argued that characterizing estrogen solely as an anabolic hormone in muscle tissue is overly simplistic. Previous research involving female rats has indicated that estrogen may also play a role in protein breakdown [38]. We also report that estradiol showed a significant association with stabilizing muscle area rather than locomotor muscle area. One plausible explanation could be that abdominal stabilizing muscles, which are associated with stability and muscular endurance, contains more type I (slow-twitch) muscle fibers [39]. Studies have demonstrated that type I fibers contain more adipose tissue and are the primary fiber type in women [40,41].

Secondly, we observed strong but non-significant associations between total testosterone and abdominal muscle composition. This is in accordance with earlier systematic reviews [12]. However, free testosterone was positively associated with abdominal muscle area. In accordance with our findings, van Geel et al., observed an inverse correlation for bioavailable testosterone and age-related loss of lean body mass and thigh strength in post-menopausal women [13]. Similar results were shown in a longitudinal study in elderly women between *free* testosterone and lean mass measured 11–16 years later by DEXA scans [14]. Plausible mechanisms by which testosterone may enhance lean mass in postmenopausal women include an increase in muscle protein synthesis, increasing structural proteins in myocyte, improving skeletal muscle function and inhibiting regulators of muscle protein degradation [42,43]. Other potential mediators in the relationship between testosterone and muscle mass in women may include insulin, which mediates the connection between testosterone and regulators of muscle protein degradation; the indirect effects of testosterone through its conversion to estrogen; and SHBG, which plays a key role in transporting testosterone in the bloodstream [43–45]. However, further investigation is needed to fully understand the relationship between free testosterone and muscle characteristics in postmenopausal women. This includes examining endogenous testosterone levels and utilizing imaging techniques such as CT or MRI for more accurate assessments.

Finally, while the bioactive role of *SHBG* remains a subject of ongoing research, studies have reported paradoxical associations related to *SHBG* and body composition. Our study revealed an independent relationship between elevated *SHBG* levels and lower abdominal muscle mass and radiodensity. Similar results have been observed in men [17]. This is in concurrence with earlier reports, presenting an inverse longitudinal association between SHBG and lean mass in postmenopausal women [14]. In contrast to our findings, Arathimos et al. demonstrated a positive relationship between *SHBG* and both lean mass and abdominal adipose mass [46]. However, in accordance with our results, earlier findings have shown consistent inverse associations for SHBG with abdominal adipose, insulin resistance and type 2 diabetes mellitus, in post-menopausal women [47]. Nevertheless, other factors influencing this relationship should be investigated, including nutritional status. For example, individuals with overweight/obesity on a low fat or glycemic diet, may exhibit elevated SHBG levels [48]. Moreover, elevated SHBG levels may potentially influence the lipolytic effects of bioactive testosterone and estrogen on ectopic adipose deposition, leading to increased myosteatosis. While the link between SHBG and muscle composition warrants further investigation, previous studies have highlighted SHBG’s biological role in mediating sex disparities and glucose metabolism. The KORA study reported higher SHBG levels in women compared to men, which may contribute to observed differences in glucose metabolism and type 2 diabetes risk [49]. Notably, SHBG appears to exert an active biological role, independent of sex hormones, and is associated with lower fasting glucose in women, potentially through interactions with muscle tissue, the main tissue responsible for glucose homeostasis [50]. These findings underscore the importance of considering SHBG as a key factor in understanding sex-related differences in metabolic health. Interestingly, the association between SHBG and abdominal muscle radiodensity became non-significant when adjustment was made for BMI. This highlights the complexity of body composition assessment. Unlike BMI, which reflects both fat and lean mass, abdominal fat represents a distinct, metabolically active depot. These findings underscore the value of precise metrics for understanding muscle and metabolic health interactions. Further examination is needed to determine the potential role of SHBG and distribution of ectopic adiposity. We employed the muscle area index (muscle area/BMI) to normalize muscle area for overall body size in our assessment of the relationship between sex hormones and muscle composition [51]. The association between SHBG and muscle area remained significant when adjusting for BMI, but it somewhat weakened when using the muscle area index. This has been a topic of debate in previous studies [51].

Strengths of this study include the use of CT-derived imaging techniques to investigate muscle characteristics in post-menopausal women. Earlier studies have reported that abdominal imaging can assess wholebody muscle and adipose status [52]. A further strength of this study included usage of data from a large and multi-ethnic cohort with detailed collection of information and blood samples, using validated instruments. The primary limitation of this study included the assessment of sex hormones, using radioimmunoassay (RIA) techniques which have been shown to overestimate estradiol and testosterone concentrations [53]. We recommend that future studies incorporate mass spectrometry for the measurement of sex hormone levels to enhance accuracy and reliability [54]. Also, free testosterone was calculated rather than directly measured, which is known to yield higher estimates [55]. The Sodergard method, while commonly used for calculating free testosterone in endocrinology, has limitations to include assuming a fixed albumin concentration, producing higher estimates than other algorithms, and being most accurate only when competing steroids are minimal and SHBG levels are within the normal range [56].Another limitation was in the timing of data collection, with sex hormones measured at visit 1 while CT-scans were obtained 2–5 years later during visit 2 or visit 3. We partially addressed this limitation by adjusting for the time lapse from baseline to the CT-scans, which did not significantly affect the results. Additionally, because different CT imaging scanners were used, the energy levels of the X-ray beams varied between scans (ranging from 120 to 140 kVp), which represents an additional limitation. However, studies in MESA have shown comparable reproducibility between electron-beam and multidetector scanners for CAC measurements, with high agreement rates [57,58]. Furthermore, all scans in our study were processed with the same software, showing inter- and intrareader reliabilities of 0.99 for abdominal, subcutaneous, and visceral areas, and 0.93 to 0.98 for muscle groups. Lastly, the observational and cross-sectional design of the study limits its ability to establish causality for the observed associations and makes it susceptible to residual confounding, as well as potential temporal and selection biases.

## 5. Conclusion

In this study, we examined the independent associations between sex hormones and abdominal muscle characteristics in post-menopausal women. Notably, we identified significant and positive associations between estradiol and *free* testosterone levels with abdominal muscle area, emphasizing their potential role in preserving muscle health. Conversely, our findings reveal an independent and inverse association between SHBG and both abdominal muscle area and radiodensity, shedding light on the nuanced interplay of hormones in muscle composition. We advocate for further prospective observational studies to investigate these relationships, contributing to a better understanding of age-related changes in muscle composition in post-menopausal women.

## Supplementary Material

1

## Figures and Tables

**Table 1:** Baseline characteristics of the study population.

Postmenopausal women (N = 682)	Mean ± SD/N (%)
Age (years)	63.3 (±9.0)
Age range	
45–54 years	149 (22 %)
54–64 years	218 (32 %)
65–74 years	218 (32 %)
75–84 years	97 (14 %)
SBP (mm Hg)	128.4 (±23.5)
DBP (mm Hg)	69.5 (±10.1)
Total abdominal muscle area (cm^2^)	80.0 (±16.8)
Total abdominal muscle area index (cm^2^/BMI)	3.0 (±0.7)
Total abdominal muscle radiodensity (HU)	40 (±5.0)
Abdominal stabilizing muscle area (cm^2^)	61.8 (±14.6)
Abdominal stabilizing muscle radiodensity (HU)	37.03 (±5.5)
Abdominal locomotor muscle area (cm^2^)	18.2 (±3.6)
Abdominal locomotor muscle radiodensity (HU)	49.9 (±5.0)
Total abdominal adipose area (cm^2^)	450.2 (±164.9)
Subcutaneous adipose area (cm^2^)	296.8 (±121.8)
Visceral adipose area (cm^2^)	124.4 (±57.5)
Waist circumference (cm^2^)	94.2 (±14.1)
WHR	0.9 (±0.1)
BMI (kg/m^2^)	27.4 (±5.2)
hsCRP (mg/L)	3.8 (±5.4)
TT (nmol/L)	1.0 (±0.7)
fT (pmol/L)	13.8 (±14.9)
SHBG (nmol/L)	79.6 (±57.2)
Estradiol (pmol/L)	143.8 (±181.4)
Race/ethnicity	
Non-Hispanic White	269 (39 %)
Chinese American	100 (15 %)
Black	150 (22 %)
Hispanic/Latino	163 (24 %)
Time from baseline to CT (years)	2.8 (±0.9)
Sedentary behavior (MET-hours/week)	29.32 (±19.3)
Total intentional exercise (MET-hours/week)	24.2 (±33.18)
Current cigarette smokers	77 (11 %)
Current alcohol consumption	476 (68 %)
Diabetes mellitus	72 (10 %)
Hypertension	340 (49 %)
Dyslipidemia	207 (30 %)
Thyroid agents use	70 (10 %)
Exogenous estrogen use	234 (33 %)
Years since menopause	15.6 (±10.7)

SBP (systolic blood pressure), DBP (diastolic blood pressure), BMI (body mass index), WHR (waist-hip-ratio), hsCRP (high sensitivity c-reactive protein), SHBG (sex hormone binding globulin), fT (free testosterone), TT (total testosterone).

**Table 2:** The associations between total and free testosterone, SHBG and estradiol with measurements of total abdominal muscle area in postmenopausal women.

Total abdominal muscle area							
Testosterone		Free testosterone		SHBG		Estradiol	
β	95 % CI	β	95 % CI	β	95 % CI	β	95 % CI
Model 1							
**5.99**	−2.5, 14.5	**11.78**	1.6, 21.9	**−1.46**	−2.3, −0.6	**0.73**	−0.1, 1.5
Model 2							
**8.40**	−3.9, 20.7	**21.7**	8.7, 34.8	**−1.89**	−2.8, −1.0	**1.75**	0.8, 2.7
Model 3							
**11.26**	−1.7, 24.2	**18.5**	4.0 33.1	**−2.1**	−3.2, −0.9	**1.41**	0.4, 2.4

Linear regressions are used to investigate the associations in three models. The magnitudes of the associations were quantified as one-unit increment of the distribution of testosterone (total and free), estradiol and SHBG, with an increase in HU for abdominal muscle radiodensity. Model 1 adjusted for age, race/ethnicity, and level of education. Model 2 included variables from model 1 along with SHBG (no adjustment was made when investigating the associations of free testosterone and SHBG), and total abdominal adipose tissue. Model 3 incorporated variables from model 2 with additional adjustments for DHEA, CRP, physical activity, sedentary behavior, cigarette smoking, alcohol consumption, time from baseline to CT, years in menopause, hypertension, diabetes mellitus, dyslipidemia, exogenous estrogen use (excluding vaginal creams) and thyroid agents β = standardized coefficient, defined as the change in HU for muscle radiodensity and cm2 for abdominal muscle area for each 1-SD increment change in levels of testosterone, estradiol and SHBG.

**Table 3:** The associations between total and free testosterone, SHBG and estradiol with measurements of total abdominal muscle index in postmenopausal women.

Total abdominal muscle area index							
Testosterone		Free testosterone		SHBG		Estradiol	
β	95 % CI	β	95 % CI	β	95 % CI	β	95 % CI
Model 1							
**−0.35**	−0.7, 0.0	**−0.66**	−1.12, −0.2	**0.07**	0.0, 0.1	**−0.0**	−0.0, 0.0
Model 2							
**−0.11**	−0.5, 0.3	**0.14**	−0.3, 0.6	**−0.02**	−0.1, 0.0	**0.03**	−0.0, 0.1
Model 3							
**−0.08**	−0.5, 0.4	**0.13**	−0.4, 0.6	**−0.03**	−0.1, 0.0	**0.02**	−0.0, 0.1

Linear regressions are used to investigate the associations in three models. The magnitudes of the associations were quantified as one-unit increment of the distribution of testosterone (total and free), estradiol and SHBG, with an increase in HU for abdominal muscle radiodensity. Model 1 adjusted for age, race/ethnicity, and level of education. Model 2 included variables from model 1 along with SHBG (no adjustment was made when investigating the associations of free testosterone and SHBG), and total abdominal adipose tissue. Model 3 incorporated variables from model 2 with additional adjustments for DHEA, CRP, physical activity, sedentary behavior, cigarette smoking, alcohol consumption, time from baseline to CT, years in menopause, hypertension, diabetes mellitus, dyslipidemia, exogenous estrogen use (excluding vaginal creams) and thyroid agents β = standardized coefficient, defined as the change in HU for muscle radiodensity and cm2 for abdominal muscle area for each 1-SD increment change in levels of testosterone, estradiol and SHBG.

**Table 4:** The associations between total and free testosterone, SHBG and estradiol with measurements of total abdominal muscle radiodensity in postmenopausal women.

Total abdominal muscle radiodensity							
Testosterone		Free testosterone		SHBG		Estradiol	
β	95 % CI	β	95 % CI	β	95 % CI	β	95 % CI
Model 1							
**−1.96**	−4.4, 0.5	**−1.7**	−4.6, 1.3	**0.14**	−0.1, 0.4	**−0.09**	−0.3, 0.1
Model 2							
**−0.07**	−3.5, 3.3	**2.46**	−1.1, 6.0	**−0.22**	−0.5, 0.0	**0.12**	−0.1, 0.4
Model 3							
**0.65**	−2.9, 4.2	**2.73**	−1.2, 6.6	**−0.32**	−0.6, −0.0	**0.03**	−0.3, 0.3

Linear regressions are used to investigate the associations in three models. The magnitudes of the associations were quantified as one-unit increment of the distribution of testosterone (total and free), estradiol and SHBG, with an increase in HU for abdominal muscle radiodensity. Model 1 adjusted for age, race/ethnicity, and level of education. Model 2 included variables from model 1 along with SHBG (no adjustment was made when investigating the associations of free testosterone and SHBG), and total abdominal adipose tissue. Model 3 incorporated variables from model 2 with additional adjustments for DHEA, CRP, physical activity, sedentary behavior, cigarette smoking, alcohol consumption, time from baseline to CT, years in menopause, hypertension, diabetes mellitus, dyslipidemia, exogenous estrogen use (excluding vaginal creams) and thyroid agents β = standardized coefficient, defined as the change in HU for muscle radiodensity and cm2 for abdominal muscle area for each 1-SD increment change in levels of testosterone, estradiol and SHBG.

## Data Availability

There are no linked research data sets for this paper. The supporting data for the conclusions drawn in this study can be obtained from the MESA committee, but access is subject to certain restrictions as they were utilized under license for the present study and are not publicly accessible. Nevertheless, interested parties can request access to the data directly from the authors, pending approval from the MESA committee.

## Appendix A. Supplementary data

Supplementary data to this article can be found online at https://doi.org/10.1016/j.maturitas.2025.108197.
