# Supplementary material for: Exploring the Relationships between Sex Hormones and Abdominal Muscle Area and Radiodensity in Postmenopausal Women: Insights from the Multi-Ethnic Study of Atherosclerosis
Source: Maturitas. Author manuscript; Available in PMC 2026 Jun 28. (PMC13310363; doi:10.1016/j.maturitas.2025.108197)
Supplement: 1 [file NIHMS2171826-supplement-1.docx]

**Supplementary Tables**

**Table 5.** The association between total and free testosterone, SHBG and estradiol with measurements of stabilizing abdominal muscles in postmenopausal women, MESA-study.

| Stabilizing Muscle Area | | | | | | | |
| --- | --- | --- | --- | --- | --- | --- | --- |
| Testosterone | | Free Testosterone | | SHBG | | Estradiol | |
| β | 95 % CI | β | 95 % CI | β | 95 % CI | β | 95 % CI |
|  | | | | | | | |
| 5.35 | -2.0, 12.7 | 11.03 | 2.2, 19.8 | -1.61 | -2.5, -0.7 | 0.90 | -0.0, 1.8 |
|  | | | | | | | |
| 5.62 | -5.1, 16.3 | 18.42 | 7.0, 29.8 | -2.09 | -3.1, -1.1 | 2.14 | 1.0, 3.2 |
|  | | | | | | | |
| 6.90 | -4.4, 18.3 | 15.7 | 3.4, 28.0 | -1.70 | -2.7, -0.7 | 1.27 | 0.4, 2.2 |

| Stabilizing Muscle Area Index | | | | | | | |
| --- | --- | --- | --- | --- | --- | --- | --- |
| Testosterone | | Free Testosterone | | SHBG | | Estradiol | |
| β | 95 % CI | β | 95 % CI | β | 95 % CI | β | 95 % CI |
|  | | | | | | | |
| -0.1 | -4.4, 4.2 | 1.03 | -4.1, 6.2 | 0.06 | 0.0, 0.1 | -0.01 | -0.0, 0.0 |
|  | | | | | | | |
| 1.53 | -4.2, 7.3 | 9.46 | 3.3, 15.6 | -0.03 | -0.1, 0.0 | 0.04 | -0.0, 0.1 |
|  | | | | | | | |
| 1.60 | -4.6, 7.7 | 6.5 | -0.1, 13.2 | -0.84 | -1.4, -0.3 | 0.78 | 0.3, 1.3 |

| Stabilizing Muscle Radiodensity | | | | | | | |
| --- | --- | --- | --- | --- | --- | --- | --- |
| Testosterone | | Free Testosterone | | SHBG | | Estradiol | |
| β | 95 % CI | β | 95 % CI | β | 95 % CI | β | 95 % CI |
|  | | | | | | | |
| -1.67 | -4.4, 1.0 | -1.02 | -4.2, 2.2 | 0.23 | -0.1, 0.6 | -0.05 | -0.4, 0.3 |
|  | | | | | | | |
| 0.63 | -3.1, 4.4 | 2.9 | -1.0, 6.9 | -0.18 | -0.5, 0.2 | 0.23 | -0.2, 0.6 |
|  | | | | | | | |
| 1.23 | -2.8, 5.2 | 3.25 | -1.1, 7.6 | -0.25 | -0.6, 0.1 | 0.11 | -0.3, 0.5 |

Linear regressions are used to investigate the associations in three models. The magnitudes of the associations were quantified as one-unit increment of the distribution of testosterone (total and free), estradiol and SHBG, with an increase in HU for abdominal muscle radiodensity. Model 1 adjusted for age, race/ethnicity, and level of education. Model 2 included variables from model 1 along with SHBG (no adjustment was made when investigating the associations of free testosterone and SHBG), and total abdominal adipose tissue. Model 3 incorporated variables from model 2 with additional adjustments for DHEA, CRP, physical activity, sedentary behavior, cigarette smoking, alcohol consumption, time from baseline to CT, years in menopause, hypertension, diabetes mellitus, dyslipidemia, exogenous estrogen use (excluding vaginal creams) and thyroid agents β (unstandardized coefficient of the association), 95 % CI (95 % Confidence Interval).

**Table 6.** The association between total and free testosterone, SHBG and estradiol with measurements of locomotor abdominal muscles in postmenopausal women, MESA-study.

| Locomotor Muscle Area | | | | | | | |
| --- | --- | --- | --- | --- | --- | --- | --- |
| Testosterone | | Free Testosterone | | SHBG | | Estradiol | |
| β | 95 % CI | β | 95 % CI | β | 95 % CI | β | 95 % CI |
|  | | | | | | | |
| 0.45 | -1.5, 2.4 | 0.75 | -1.6, 3.1 | -0.27 | -0.5, -0.0 | 0.08 | -0.2, 0.3 |
|  | | | | | | | |
| 2.77 | -0.0, 5.6 | 3.31 | 0.3, 6.3 | -0.25 | -0.5, -0.1 | 0.25 | -0.0, 0.5 |
|  | | | | | | | |
| 4.36 | 1.4, 7.4 | 4.30 | 1.0, 7.6 | -0.42 | -0.7, -0.2 | 0.12 | -0.1, 0.4 |

| Locomotor Muscle Area Index | | | | | | | |
| --- | --- | --- | --- | --- | --- | --- | --- |
| Testosterone | | Free Testosterone | | SHBG | | Estradiol | |
| β | 95 % CI | β | 95 % CI | β | 95 % CI | β | 95 % CI |
|  | | | | | | | |
| -0.42 | -1.5, 0.7 | -0.93 | -2.3, 0.4 | 0.03 | 0.0, 0.0 | -0.01 | -0.0, 0.0 |
|  | | | | | | | |
| 1.19 | -0.3, 2.7 | 1.74 | 0.19, 3.3 | 0.00 | -0.0, 0.0 | -0.00 | -0.0, 0.0 |
|  | | | | | | | |
| 1.86 | 0.3, 3.4 | 1.91 | 0.2, 3.6 | -0.2 | -0.3, -0.1 | 0.08 | -0.0, 0.2 |

| Locomotor Muscle Radiodensity | | | | | | | |
| --- | --- | --- | --- | --- | --- | --- | --- |
| Testosterone | | Free Testosterone | | SHBG | | Estradiol | |
| β | 95 % CI | β | 95 % CI | β | 95 % CI | β | 95 % CI |
|  | | | | | | | |
| -2.87 | -5.7, -0.0 | -3.5 | -6.9, -0.1 | 0.07 | -0.3, 0.4 | -0.28 | -0.6, 0.1 |
|  | | | | | | | |
| -2.14 | -5.8, 1.5 | 1.04 | -2.9, 5.0 | -0.46 | -0.7, -0.2 | -0.01 | -0.4, 0.4 |
|  | | | | | | | |
| -0.27 | -0.7, 0.20 | 1.16 | -3.1, 5.4 | -0.53 | -0.9, -0.2 | -0.16 | -0.5, 0.1 |

Linear regressions are used to investigate the associations in three models. The magnitudes of the associations were quantified as one-unit increment of the distribution of testosterone (total and free), estradiol and SHBG, with an increase in HU for abdominal muscle radiodensity. Model 1 adjusted for age, race/ethnicity, and level of education. Model 2 included variables from model 1 along with SHBG (no adjustment was made when investigating the associations of free testosterone and SHBG), and total abdominal adipose tissue. Model 3 incorporated variables from model 2 with additional adjustments for DHEA, CRP, physical activity, sedentary behavior, cigarette smoking, alcohol consumption, time from baseline to CT, years in menopause, hypertension, diabetes mellitus, dyslipidemia, exogenous estrogen use (excluding vaginal creams) and thyroid agents β (unstandardized coefficient of the association), 95 % CI (95 % Confidence Interval).
